# Marginal and internal fit of crowns based on additive or subtractive manufacturing

**DOI:** 10.1080/26415275.2021.1938576

**Published:** 2021-06-26

**Authors:** Yasser Haddadi, Bahram Ranjkesh, Flemming Isidor, Golnosh Bahrami

**Affiliations:** Department of Dentistry and Oral Health, Section for Prosthodontics, Aarhus University, Aarhus, Denmark

**Keywords:** Additive manufacturing, 3D printing, digital dentistry, marginal fit, internal fit, fit of crowns

## Abstract

**Objective:**

To assess the marginal and internal fit of crowns manufactured by additive and subtractive manufacturing technique.

**Materials and Methods:**

Twenty extracted teeth prepared for complete coverage crowns were scanned with an intra-oral scanner (Omnicam, DentsplySirona). For the subtractive manufacturing (SM) group, ten crowns were manufactured in a hybrid resin block (Vita Enamic, Vita Zahnfabrik). For the additive manufacturing (AM) group, the crowns were manufactured in a hybrid resin material (NextDent C&B, 3D systems). The design parameters were identical for the two groups. The marginal and internal fit (determined at the axial wall, the cusp tip and occlusally) was assessed before cementation with the replica technique and after cementation under stereomicroscope after sectioning of the crowned teeth.

**Results:**

For the SM group, the marginal fit was 91 µm (±28 µm) before cementation and 85 µm (±18 µm) after cementation. In the AM group, the marginal fit was 75 µm (±29 µm) before cementation and 71 µm (±18 µm) after cementation. The differences were not statistically significant. As regards the internal fit, the fit at the axial wall was statistically significantly better in the SM group than in the AM group (*p*=.009 before cementation and .03 after cementation). Occlusally the fit in the AM group was significantly better than in the SM group after cementation (*p*<.001).

**Conclusion:**

Within the limitations of the current study, the marginal fit of additively manufactured crowns is comparable to crowns manufactured with chair-side subtractive technique and within the clinically acceptable range. As regards the internal fit no one technique was consistently superior.

## Introduction

The workflows in dentistry, and particularly in fixed prosthodontics, are increasingly shifting towards automation and digitization [1,2]. A fully digital workflow starts with digitizing the oral hard and soft tissues with an intra-oral scanner, then designing a restoration using computer-aided design (CAD) software and subsequently manufacturing a restoration by computer-aided manufacturing (CAM).

Well-established intra-oral scanners have been shown to perform equal to, if not better than conventional impression methods with addition-cured silicone materials, particularly for shorter spans such as a single tooth, up to a quadrant [3–5]. The manufacturing phase for a fixed prosthetic restoration, after a scan is obtained and a restoration has been designed, is currently subtractive manufacturing (SM), i.e. milling, where the final restoration is milled out of a block. There are a wide range of materials that can be used in this manufacturing process, which can be performed chair-side within a very limited time frame compared to the traditional manufacturing methods. The scan can also be sent to a technician for the production of a restoration. The sufficient accuracy of the current digital workflow for SM of single unit crowns is well established both *in vitro* and *in vivo* [6–11].

In recent years, the different methods of additive manufacturing (AM) have evolved and several of these have great potential in dentistry [12,13]. Selective laser melting (SLM) and direct metal laser sintering (DMLS) are methods of using AM for the production of crowns in different metal alloys [14,15].

For chair-side dentistry, the rapid developments in photopolymerization, i.e. stereolithography (SLA) and digital light projection (DLP), and the available resins have been of particular interest. SLA and DLP are methods where laser or ultraviolet light is used to illuminate and polymerize the liquid resin contained in a vat. This is repeated numerous times to finally produce a three-dimensional (3D) structure [16]. AM using SLA or DLP has been used for the production of dental models, splints and surgical guides with short-term intra-oral application time [16,17]. Interestingly, resins and hybrid ceramic resins have recently been developed and approved by the FDA as grade IIa materials for long-term intra-oral use, thereby making it possible to additively manufacture long-term interim hybrid resin fixed prosthetic crowns. However, there is limited literature available on the accuracy of crowns manufactured chair-side using AM, compared to SM. The purpose of the present study was to investigate the marginal and internal fit of single-unit crowns manufactured by AM compared to SM, using a chairside CAD-CAM system.

H0: There is no difference in the marginal or internal fit of crowns before and after cementation manufactured using additive or subtractive manufacturing.

## Materials and methods

Twenty caries and restoration-free, extracted third molars were prepared for complete coverage crowns with a smooth circumferential accentuated chamfer. The teeth were numbered and a person not involved in the preparation of the teeth performed a closed randomization and divided the teeth into two groups (*n* = 10). All of the prepared teeth were scanned using an Omnicam intra-oral scanner (DentsplySirona) with software version 4.5.2. Care was taken to ensure sufficient data capture of all tooth surfaces and preparation margins.

The crowns in the SM group were designed in CEREC software version 4.5.2 and milled using an MCX milling station (DentsplySirona) in a hybrid resin block (Vita Enamic, Vita Zahnfabrik). The milling station was calibrated prior to use, and before milling new burs, a 12S step bur and a 12S cylinder pointed bur were installed. To improve accuracy, the fine mode of milling was used. The cement gap at the margin was set at 0.020 mm; 1.2 mm from the margin line, the cement gap gradually increased to 0.1 mm. No further post-processing steps were undertaken after milling.

In the AM group, the crowns were designed in appropriate design software (Inlab, software version 19, DentsplySirona) using the same settings as for the SM group and manufactured with a DLP printer (NextDent 5100, 3D Systems) with a hybrid resin material (NextDent C&B, 3D systems). The smallest possible layer increments were used (0.025 mm). A build angle of 135 degrees was chosen, as this has been shown to improve print accuracy [18]. After manufacturing, the recommended wash and post-print polymerization procedure was performed for the printed crowns, according to the manufacturer’s instruction.

**Figure 1.**
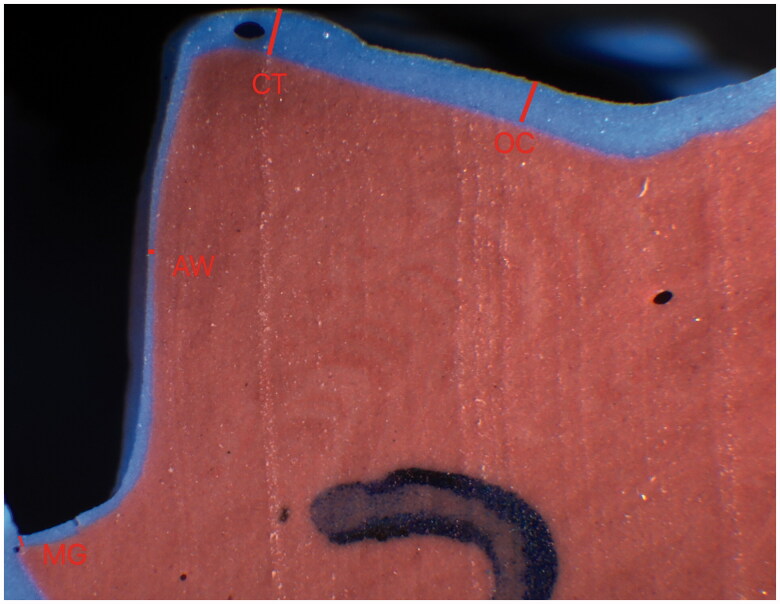
Sectioned replica. Blue silicone material represents space between tooth and crown. Margin (MG), axial wall (AW), cusp tip (CT) and occlusal surface (OC).

The marginal and internal fit of the crowns were measured before cementation using the replica technique, and after cementation by sectioning the crown and tooth. In order to measure the marginal and internal fit of the crowns before cementation, silicone replicas of the gap between the abutment teeth and the crowns was obtained using the replica technique, as described by Molin and Karlson [19], and Boening et al. [20], Thus, all crowns were fully seated using firm finger pressure, with light-body silicone (Extrude, Kerr) applied internally in the crowns. After setting of the light-bodied material, the internal impression was stabilized with a heavy-body material (Extrude, Kerr) in a different colour. The light-body replica impression was assessed under loupes (2.3× magnification) and if any defects were observed, the impression was retaken. A scalpel (10 A, Swann Morton) was used to section the replicas. Replicas were sectioned in mesiodistal and buccolingual directions, resulting in four cross-sections for each restoration. The span of the cement space corresponding to light-body silicone material thickness was measured at four predetermined locations. Marginal fit was assessed as the marginal gap (MG) as defined by Holmes et al. [21]. The internal fit was assessed at three different points; the axial wall (AW), the cusp tip (CT), and the occlusal surface (OC) on each cross-section (Figure 1), resulting in 16 measurements per replica.

**Figure 2.**
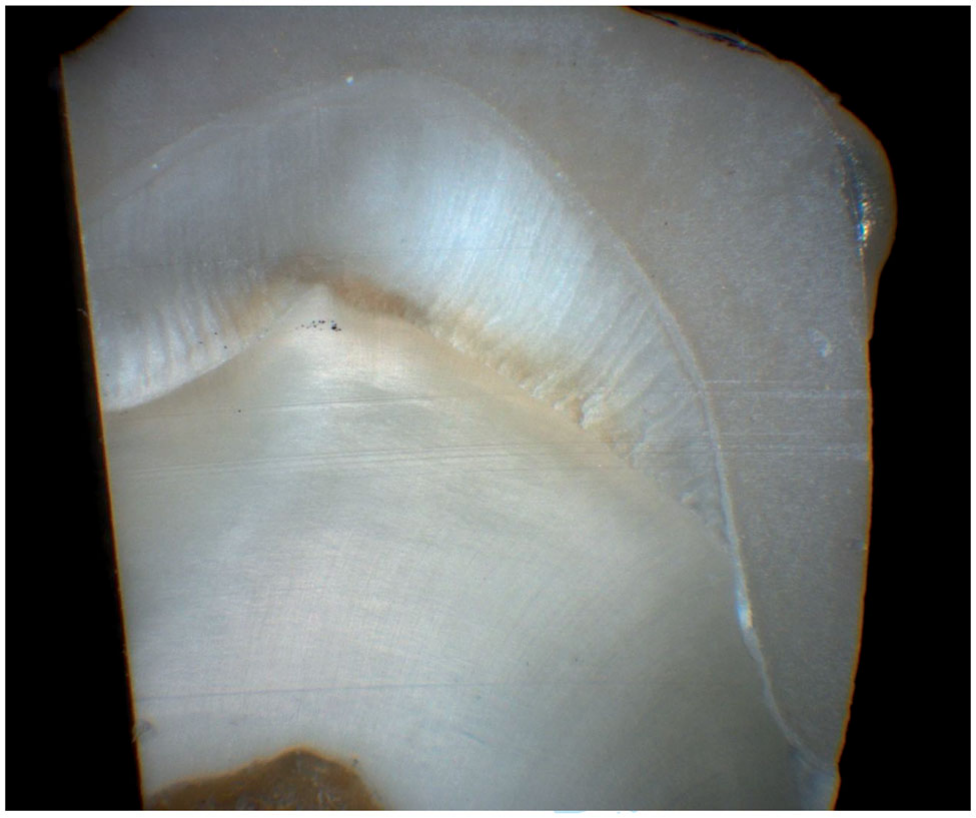
Sectioned tooth after cementation.

After the replica technique was performed, all crowns were cemented on their corresponding teeth using a dual-cure resin cement (Multilink Automix, IvoclarVivadent). Once the crowns were cemented, the teeth were sectioned in mesiodistal and buccolingual directions similar to the silicone replicas (Figure 2). The sectioning was performed with a microtome saw with a 0.3 mm blade (Leica 1600, Leica Biosystems). Landmarks were used in an attempt to section the crowns near the place of replica sections. For each sectioned tooth 16 measurements were performed. The marginal and internal fit for both groups was linearly measured using a stereomicroscope (Wild Macroscope M420, Wild) and digital camera (Zeiss AxioCam MRc5, Carl Zeiss Micro Imaging GmbH) with 40× magnification on the computer screen.

### Statistical analysis

The mean and standard deviation (SD) were calculated for each tooth and then for each group before and after cementation. Data were tested for normality using QQ plots. As the data were normally distributed, a two-way analysis of variance (ANOVA) followed by pairwise comparisons with *post hoc* Tukey’s were performed to detect statistically significant differences between the groups. The significance level was set at .05. Data were analysed using appropriate software (STATA 14, STATACORP).

## Results

Table 1 displays the results for the marginal and internal fit for both groups.

**Table 1.** Marginal fit and internal fit at the axial wall, cusp tip and occlusal point of crowns manufactured using subtractive or additive techniques before and after cementation (means values and standard deviations in µm).

Measuring timepoint	Group	Marginal fit (µm)	Axial wall (µm)	Cusp tip (µm)	Occlusal point (µm)
Before cementation (replica)	Subtractive manufacturing	91 (28)	120 (22)^a^	353 (137)	322 (55)^a^
	Additive manufacturing	75 (29)	145 (17)^b^	253 (86)	237 (39)^b^
After cementation (sectioning)	Subtractive manufacturing	85 (18)	117 (39)^a^	271 (82)	227 (45)
	Additive manufacturing	71 (18)	136 (44) ^b^	222 (51)	218 (56)

Different letters indicate statistically significant difference in the results.

The difference between the groups at the margin was not statistically significant regardless of the measuring timepoint.

For the internal gaps, the axial wall results for SM were statistically significantly lower than for AM for both measuring timepoints, indicating a better fit of the SM than the AM crowns (before cementation: *p*=.009; after cementation: *p*=.03). The difference at the occlusal point was statistically significant before cementation (*p* < 0.001) but not after cementation, with the AM group displaying a better fit (237 µm (±39 µm)) than SM group (322 µm (±55 µm)).

## Discussion

The null hypothesis was rejected for the internal fit, as there was a statistically significant difference between the two manufacturing methods at the axial wall and occlusal surface. However, the results also showed that the marginal fit of AM crowns was comparable to chair-side SM crowns, and that both manufacturing methods resulted in marginal fits that are clinically acceptable according to McLean and Fraunhofer [22].

Evaluating the marginal fit of crowns can be challenging. Although the replica technique has been validated for the evaluation of the marginal and internal fit of crowns [23–25], it does have some shortcomings and can be technique-sensitive. One of the shortcomings of this technique is a limited number of measurement points for each restoration, therefore it may not represent the true circumferential fit of a crown; however, as the technique has been used extensively in prosthodontics, it makes comparisons to other studies easier. All crowns were cemented using firm finger pressure as this is a common way of seating a crown in normal dental practice. However, it is also a source of inaccuracy, as the seating pressure is not reproducible from one case to the other. After cementation, all crowns were sectioned in a similar manner to the replicas in order to increase the validity of our results, however it is not possible to ensure that the measurement are performed at the same place for both measurement techniques.

The extracted teeth were prepared free hand by an experienced dentist. Although the preparations were easy to perform and control, they were not standardized with regards to convergence angle and height of the preparation wall. A positive relationship has been shown between convergence angle and seating discrepancy [26], therefore the seating of the crowns could have been affected by the difference in preparations.

For the AM group, care was taken to ensure the optimal build angle was used to obtain the best results. After printing, an experienced technician performed all post-printing steps to ensure the geometrical integrity of the crowns were not compromised.

The field of AM in fixed prosthodontics is new and, to a large extent, unexplored. Variations in methodologies, software, printing parameters, and hardware in the current published articles make direct comparison difficult. The results of this study are, however, comparable with those of other studies evaluating the accuracy of AM crowns. Harbi et al. [27] and Bae et al. [15] found marginal and internal fits that were significantly better for AM crowns compared to SM crowns. Yet both were, as with our results, within the reported range for CAD-CAM crowns. Peng et al. [28] studied the overall internal fit of interim crowns manufactured using AM and SM. In the AM group, the same resin material as in the current study was used, but in a different printer. They found no statistically significant difference between the two groups. Mahmood et al. [29] also conducted an *in vitro* study comparing the accuracy of AM and SM crowns and they also had a conventional manufacturing group. They used the replica technique to assess the marginal and internal fit of the crowns. They concluded that the fit of digitally manufactured crowns was superior to crowns manufactured by the conventional method, and of the digital methods, the AM technique was more accurate than the SM technique.

There seems to be an trend towards a superior adaptation of AM restoration compared to SM. However, there are currently no *in vivo* studies available and, as stated earlier, the few published articles that are available use a wide range of hardware, software and methodologies.

## Conclusion

Within the limitations of the current study, the marginal fit of additively manufactured crowns was comparable to crowns manufactured with a chair-side subtractive technique and within the clinically acceptable range. As regards the internal fit no one technique was consistently superior: subtractively manufactured crowns showed better fit than additively manufactured crowns at the axial wall before as well as after cementation, but poorer fit at the occlusal point before cementation.
